# Circulating and Pulmonary T-cell Populations Driving the Immune Response in Non-HIV Immunocompromised Patients with* Pneumocystis jirovecii* Pneumonia

**DOI:** 10.7150/ijms.34512

**Published:** 2019-08-14

**Authors:** Nan-Nan Zhang, Xu Huang, Hui-Ying Feng, Lin-Na Huang, Jin-Gen Xia, Yan Wang, Yi Zhang, Xiao-Jing Wu, Min Li, Wei Cui, Qing-Yuan Zhan

**Affiliations:** 1 Center for Respiratory Diseases, China-Japan Friendship Hospital, Beijing 100029, China; 2 Department of Pulmonary and Critical Care Medicine, China-Japan Friendship Hospital, Beijing 100029, China; 3 National Clinical Research Center for Respiratory Diseases, Beijing 100029, China; 4 Graduate School of Peking Union Medical College, Chinese Academy of Medical Sciences, Beijing 100730, China; 5 Beijing Key Laboratory of Remodeling-Related Cardiovascular Diseases, Beijing Anzhen Hospital of Capital Medical University, Beijing Institute of Heart Lung and Blood Vessel Diseases, Beijing 100029, China.

**Keywords:** *Pneumocystis jirovecii* pneumonia, HIV negative, immunocompromised, T cells

## Abstract

**Background:** Previous studies in human subjects have mostly been confined to peripheral blood lymphocytes for* Pneumocystis* infection. We here aimed to compare circulating and pulmonary T-cell populations derived from human immunodeficiency virus (HIV)-uninfected immunocompromised patients with *Pneumocystis jirovecii* pneumonia (PCP) in order to direct new therapies.

**Methods:** Peripheral blood and bronchoalveolar lavage samples were collected from patients with and without PCP. Populations of Th1/Tc1, Th2/Tc2, Th9/Tc9, and Th17/Tc17 CD4^+^ and CD8^+^ T cells were quantified using multiparameter flow cytometry.

**Results:** No significant differences were found between PCP and non-PCP groups in circulating T cells. However, significantly higher proportions of pulmonary Th1 and Tc9 were observed in the PCP than in the non-PCP group. Interestingly, our data indicated that pulmonary Th1 was negatively correlated with disease severity, whereas pulmonary Tc9 displayed a positive correlation in PCP patients.

**Conclusions:** Our findings suggest that pulmonary expansion of Th1 and Tc9 subsets may play protective and detrimental roles in PCP patients, respectively. Thus, these specific T-cell subsets in the lungs may serve as targeted immunotherapies for patients with PCP.

## Introduction

*Pneumocystis jirovecii* pneumonia (PCP) is a serious infectious disease currently afflicting immunocompromised populations 1. Although the incidence of PCP in human immunodeficiency virus (HIV)-positive patients has been reduced as a consequence of improved access to effective antiretroviral therapy, the risk has increased among patients who are immunocompromised due to other causes 2, 3. For example, patients with autoimmune diseases, or who are receiving cancer chemotherapy or organ transplants are highly susceptible to severe PCP 4. The mortality rate in non-HIV patients with PCP ranges between 30 and 50%, which is significantly higher than the 7 to 17% seen in HIV-positive populations 5. An emerging concern is that increasing numbers of non-HIV patients with PCP are being admitted to intensive care units (ICU). Among this population, mortality rates have reached 58% to 80% 6-8.

There is a growing body of evidence that recognizes the critical role of the host immune response to *P. jirovecii* 9, 10. The essential role of CD4^+^ T cells in defense against *P. jirovecii* in animal models and patients is well documented 11, 12. However, CD4^+^ T cell mediated immunity may also be pathogenic, resulting in substantial lung damage during *P. jirovecii* infection by increasing inflammatory mediators, and recruiting effector cells, particularly in the process of immune reconstitution 13-15. Specific subsets of CD4^+^ T cells have been reported to mediate the immune response in animals with *P. murina* infection. In mice inoculated with *P. carinii*, Th1 and Th2 subsets were recruited into the lungs, with the response mediated predominantly by Th2 cells 16. Mice deficient in either IFN-γ or IL-4 were still able to resolve their infections 17, 18. Additionally, the IL-17/IL-23 axis was capable of extending the Th17 effector response 19, and Th17 cells are involved in the immune response during *P. jirovecii* infection 20. More recently, IL-9*^-/-^* mice showed reduced *P. murina* burden in the lungs 21. The presence of CD8^+^ T cells in response to *P. jirovecii* infection may also be deleterious 22. Of interest, it has been shown that high level Tc1-mediated production of IFN-γ could confer a protective effect that Tc2 cells do not in *P. murina*-infected animals 23, 24.

This body of research draws our attention to roles of T cells in *P. jirovecii* infection. Therefore, a better understanding of the *Pneumocystis*-related immune response might come from focusing on distinct T-cell subsets. To date, apart from a case-control study that described no significant differences in circulating T cells, including Th1, Th2, and Th17 subsets between renal transplant patients with and without PCP, studies of T-cell subset mediated immunity in PCP patients remain sparse 25. Studies of T-cell immunity in human subjects have been mostly restricted to T cells in blood. It is therefore unclear whether patients with PCP are consistent with non-PCP patients with respect to their pulmonary T-cell subsets. Thus, defining the differences between circulating and pulmonary T-cell subsets in PCP patients and non-PCP patients is critical for better understanding the pathogenic immune mechanisms.

In this study, we characterized T-cell subset profiles (including Th1, Th2, Th9, and Th17 subpopulations, as well as corresponding CD8^+^ T cells) in the blood and lungs of HIV free, immunocompromised patients, according to their *P. jirovecii* status. Clinical correlations with T-cell populations were also evaluated.

## Materials and methods

### Study subjects and sample collection

We included a total of 44 immunocompromised patients in the Department of Respiratory and Critical Care Medicine at China-Japan Friendship Hospital between March 2017 and September 2018. All patients enrolled were defined as HIV-negative with one or more of the following immunosuppressive host conditions (anti-tumor chemotherapy for solid tumor or hematologic cancer, treatments for systemic disease, or immunosuppressive therapies) 26, 27. The group was divided into two cohorts according to *P. jirovecii* status, which was determined via Giemsa and methenamine silver staining in bronchoalveolar lavage (BAL) fluid samples. Blood samples were collected and analyzed during the same period from 27 patients with PCP, and 17 patients without PCP. Concomitantly, pulmonary specimens were obtained during diagnostic procedures performed before patients initiated their therapeutic regimen. Due to the usage for clinical examination, BAL fluid samples were only obtained from 23 patients with PCP, and 14 patients without PCP. Clinical parameters for each patient were acquired through review of electronic medical records. Approval was obtained from the Ethics Committee of the China-Japan Friendship Hospital (No. 13018), and each patient provided informed consent.

### Sample processing

Fiberoptic bronchoscopy with recovery of BAL fluid was performed according to a standardized protocol 28. In brief, 5 aliquots of 40 mL saline solution were deposited into the lung lobe containing radiologic abnormalities. This solution was recollected, immediately centrifuged and placed on ice. Supernatants were discarded, and cells were resuspended in medium for flow cytometric analyses. Blood samples were prepared in heparin anticoagulant tubes by extracting peripheral blood mononuclear cells (PBMCs) isolated using Ficoll density gradient centrifugation (GE Healthcare, Sweden), and placed on the ice until analysis. Cell were washed, resuspended, and cultured in RPMI 1640 medium (Invitrogen, Carlsbad, CA, USA) containing 10% Fetal Bovine Serum (Gibco, USA).

### Multiparameter flow cytometric analysis

We used intracellular cell staining (ICS) to detect intracellular cytokines as previously reported 29. First, cells were stimulated by addition of phorbol myristate acetate (PMA; 50 ng/mL, eBioscience, San Diego, California, USA) with ionomycin (2 μg/mL, eBioscience) in the presence of brefeldin A (10 μg/mL, eBioscience) for 4 hours at 37°C. Cells were subsequently stained with extracellular anti-CD3-Percp-cy5.5, anti-CD4-APC-H7, anti-CD8-BV510 (all BD Biosciences, San Jose, California, USA) for 20 min, followed by a fixation and permeabilization procedure using Perm/Wash Buffer (BD Biosciences). Thereafter, cells were washed with permeabilization buffer and incubated with intracellular antibodies including anti-IFNγ-Alexa 488, anti- IL4-BV711, anti-IL-9-PE, and anti-IL17-BV650 (all BD Biosciences). Appropriate isotypes were used. Finally, stained cells were acquired on an LSRII Fortessa cytometer (BD Biosciences). Data were analyzed using Flowjo software (Ashland, OR, USA).

### Statistical analysis

Data management and analysis were performed using SPSS 16.0 software (Chicago, Illinois, USA). The Student's *t*-test or Mann-Whitney *U* test were used to calculate differences in continuous variables between two groups. Categorical variables were compared using χ^2^ analysis or Fisher's exact test, as appropriate. Relationships between variables were identified using Pearson or Spearson correlation, as appropriate. Data are reported as mean ± standard deviation (SD) or as median and interquartile range (IQR) (25%, 75%). Significance was defined as having a *p* value less than 0.05.

## Results

**Figure 1:**
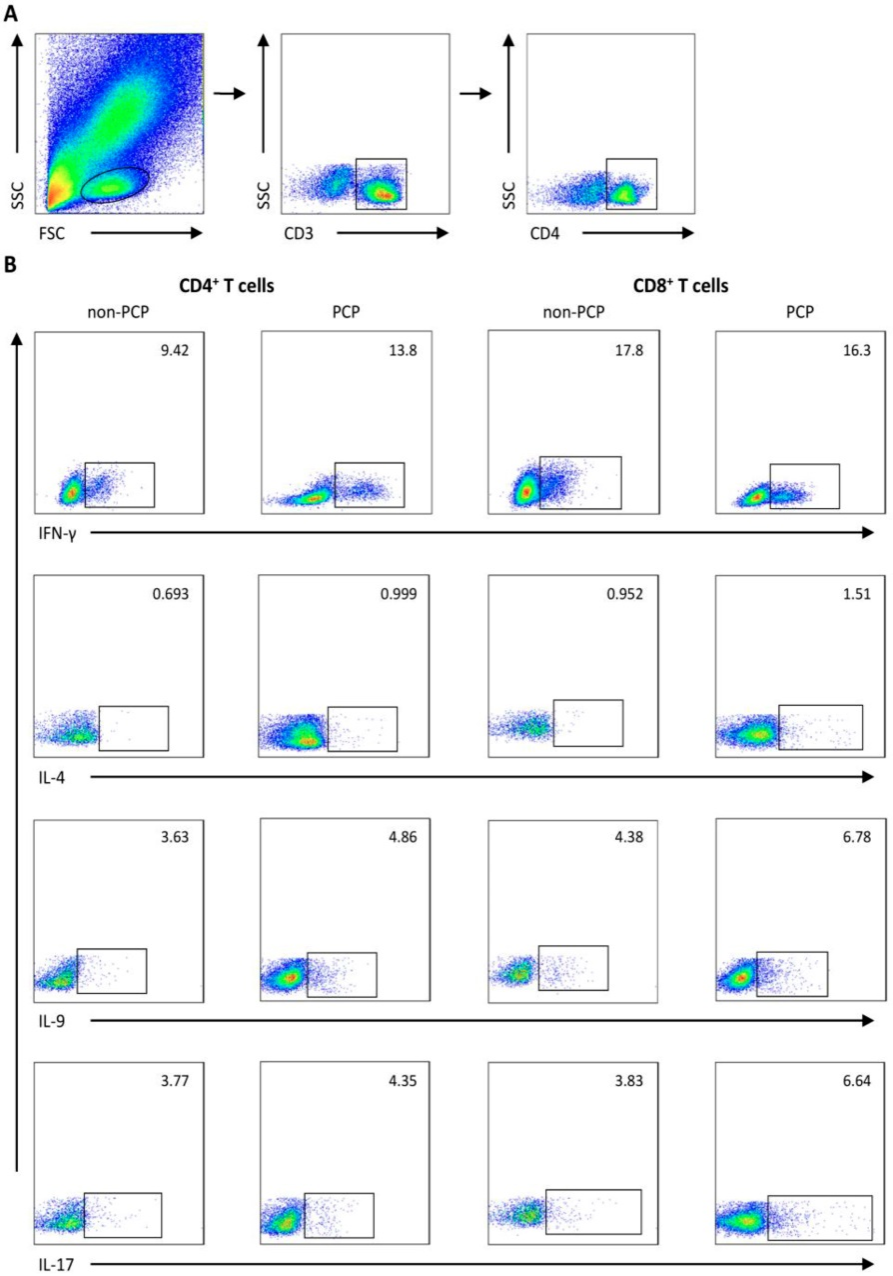
Representative flow cytometry data. Gating strategy and frequencies of T-cell subsets in blood from 1) a representative patient without *Pneumocystis jirovecii* pneumonia (PCP), and 2) a patient with PCP are shown. (**A**) Gating strategies of flow cytometric analysis. (**B**) Representative plots of subgroups of CD4^+^/CD8^+^ T cells sorted by IFN-γ, IL-4, IL-9, and IL-17A expression. FSC: forward scatter characteristics; SSC: side scatter characteristics.

We used flow cytometry to quantify IFN-γ, IL-4, IL-9 and IL-17-producing cells, which were defined as Th1/Tc1, Th2/Tc2, Th9/Tc9, Th17/Tc17 subsets of CD4^+^ and CD8^+^ T cells, respectively. Gating strategies for flow cytometry are depicted in Figure 1.

### Characteristics of study population

Twenty-seven of the total of forty-four immunocompromised patients were diagnosed with PCP. The remaining 17 were PCP free. Table 1 presents the clinical characteristics of patients in each cohort. The mean age was 57.148 ± 15.41 years in the PCP cohort, and 61.94 ± 13.069 years in the cohort without PCP, without significant difference. The sex ratios between PCP and non-PCP patients were also not different. In the patients with PCP, underlying conditions were as follows. The most common underlying immunosuppressive diseases were interstitial lung disease (48.15%), followed by systemic disease (33.33%), hematological malignancy (11.11%), solid tumor (7.41%), and solid organ transplant (7.41%). We observed no significant difference with regard to underlying diseases or the use of immunosuppressive agents between the two cohorts. Furthermore, patients in the PCP cohort were not more likely to receive prophylaxis at the time of the study than patients in the cohort without PCP.

Finally, white blood cells, lymphocyte counts, CD4^+^ T-cell counts, and CD8^+^ T-cell counts were not different between cohorts with and without PCP.

### CD4^+^ and CD8^+^ T cells in the circulation and lungs of patients with PCP

**Figure 2:**
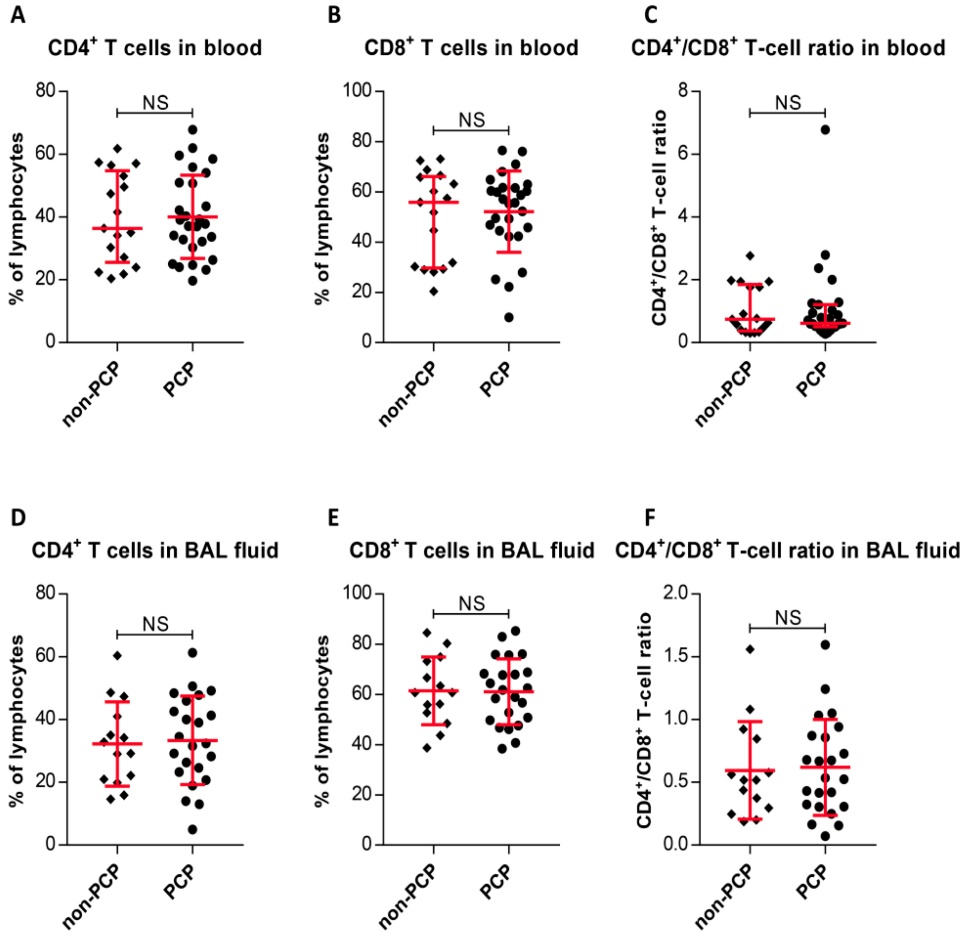
Frequencies of CD4^+^ and CD8^+^ T cells, and CD4^+^/CD8^+^ T-cell ratios. Percentages of CD4^+^ T cells (**A**) and CD8^+^ T cells (**B**), and CD4^+^/CD8^+^ T-cell ratios (**C**) in peripheral blood are exhibited. Frequencies of CD4^+^ T cells (**D**) and CD8^+^ T cells (**E**), and CD4^+^/CD8^+^ T-cell ratios (**F**) from bronchoalveolar lavage (BAL) fluid are shown. For cells from blood, n = 27 patients with *Pneumocystis jirovecii* pneumonia (PCP), and 17 patients without PCP. BAL fluid, n = 23 in PCP, and 14 patients in non-PCP groups. *P < 0.05; **P < 0.01; ***P < 0.001. NS, not significant.

We initially determined CD4^+^ and CD8^+^ T-cell frequencies in peripheral blood and BAL fluid. The results, as shown in Figure 2, indicate that PCP did not caused significant increases or decreases in CD4^+^ T-cell percentages or CD8^+^ T-cell percentages in blood relative to percentages in non-PCP (*P* < 0.0001), as were CD4^+^/CD8^+^ T-cell ratios (*P* < 0.0001, Figure 2A-C). The T-cell frequencies in BAL fluid from PCP patients were also compared with those in non-PCP patients (Figure 2D-F); it is apparent that the fractionation parameters in the blood and BAL fluid of PCP patients were similar to those in the cohort without PCP.

### T-cell subsets in the circulation of patients with PCP

**Figure 3:**
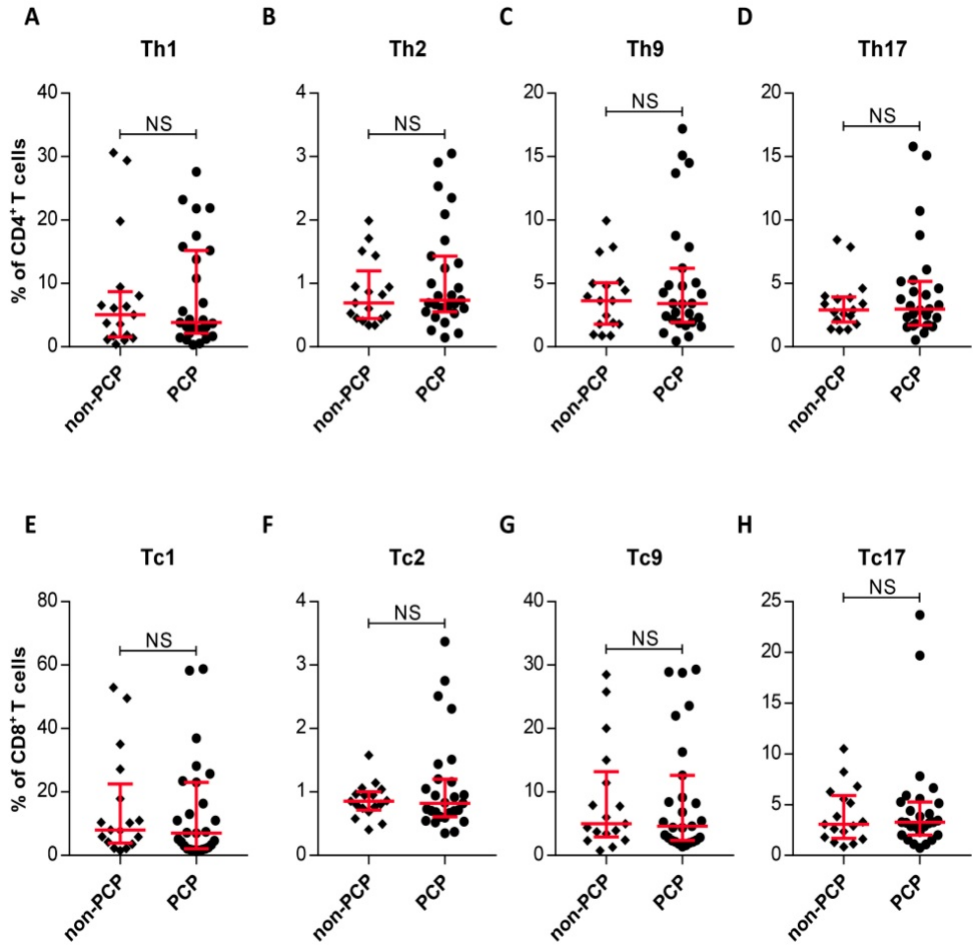
Intracellular cytokine expression in circulating CD4^+^ and CD8^+^ T cells. (**A-H**) Quantitative flow cytometric analyses of Th1/Tc1, Th2/Tc2, Th9/Tc9 and Th17/Tc17 subsets of CD4^+^/CD8^+^ T cells. Patients without *Pneumocystis jirovecii* pneumonia (PCP), n = 17; patients with PCP, n = 27. *P < 0.05; **P < 0.01; ***P < 0.001. NS, not significant.

The observation of similar frequencies of systemic and local CD4^+^, CD8^+^ T-cell from PCP and non-PCP groups prompted us to assess whether there also existed similar systemic frequencies in subsets of CD4^+^ and CD8^+^ T-cells between the two groups. As Figure 3 shows, no significant difference between the two groups was evident, as expected (Figure 3). In summary, we failed to differentiate immunocompromised patients with PCP from non-PCP patients in circulating T-cell subsets.

### Pulmonary expansion of Th1 and Tc9 in PCP cases

**Figure 4:**
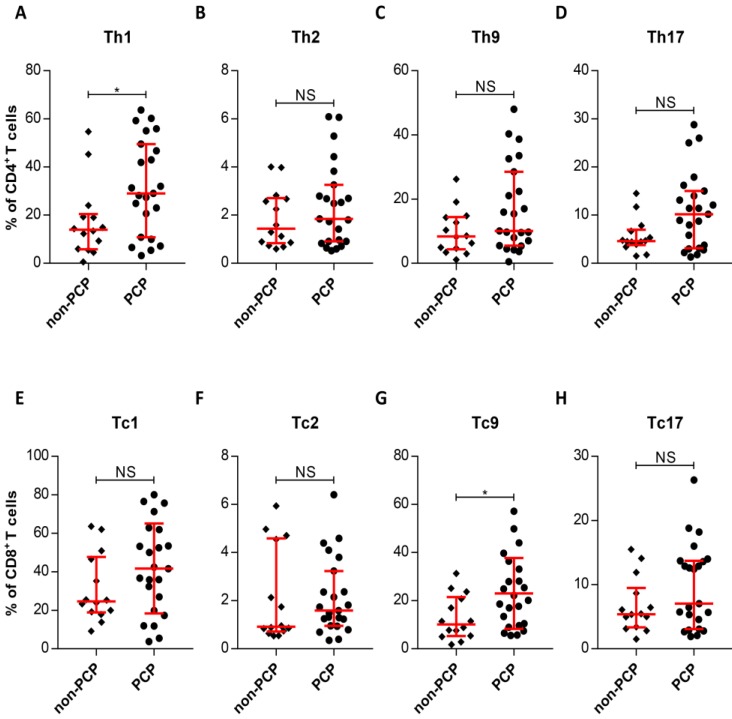
Differences in levels of pulmonary T-cell subsets. (**A-H**) Quantitation of IFN-γ^+^, IL-4^+^, IL-9^+^, and IL-17^+^ in the indicated T-cell subsets within the *Pneumocystis jirovecii* pneumonia (PCP) cohort (n = 23) and non-PCP cohort (n = 14), as indicated. *P < 0.05; **P < 0.01; ***P < 0.001. NS, not significant.

Due to the similarity in circulating T-cell subset frequencies between patients with and without PCP, we asked whether pulmonary T-cell subsets are present in similar frequencies in the two groups. We therefore compared the T-cell patterns in the lungs of patient cohorts with and without PCP. The most striking result to emerge from this data is that the frequency of pulmonary Th1 cells in PCP patients was substantially increased, compared with those in patients without PCP (*P* = 0.0199, Figure 4A). Interestingly, there was also an enhanced frequency of Tc9 cells in PCP patients, relative to patients without PCP (*P* = 0.0266, Figure 4G). Similar to results with peripheral T-cells, the T-cell subsets resident in the lungs did not differ between groups with and without PCP (Figure 4). Thus, CD4^+^ and CD8^+^ T cells in patients with PCP demonstrated local and selective enhanced frequencies of Th1 and Tc9 cells by comparison with non-PCP patients.

### Correlation between Th1 or Tc9 frequencies in the lungs and the circulating compartments, and disease severity in PCP patients

**Figure 5:**
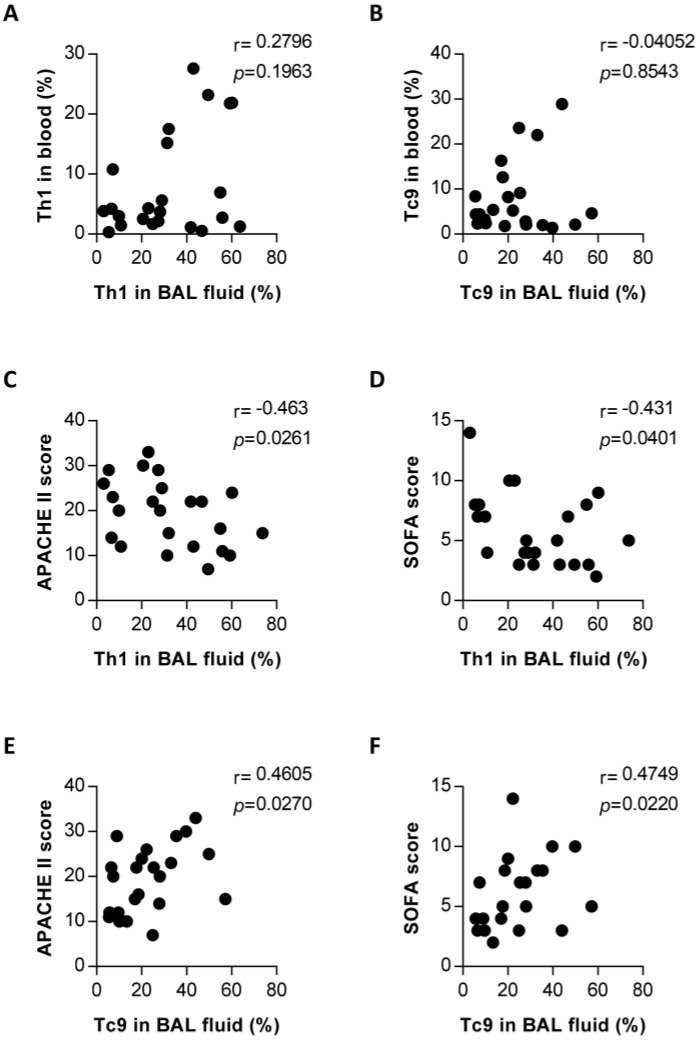
Correlation between frequencies of pulmonary Th1 or Tc9 and blood compartments, and disease severity in *Pneumocystis jirovecii* pneumonia (PCP) patients. Correlation between frequencies of pulmonary and blood Th1 (**A**) and Tc9 (**B**) in patients with PCP. Correlation between percentages of Th1 in bronchoalveolar lavage (BAL) fluid and the acute physiology and chronic health evaluation (APACHE) II score (**C**) and sequential organ failure assessment (SOFA) score (**D**). Correlation between percentages of pulmonary Tc9 and APACHE II score and SOFA score was shown in (**E**) and (**F**), respectively. n = 23. *P* < 0.05 was considered statistically significant.

Findings presented thus far suggest that Th1 and Tc9 subsets might mediate an immune response to PCP, involving expansion relative to those in patients without PCP (Figure 4). To evaluate the relationship of these upregulated T-cell subsets in the lungs with PCP, we performed clinical correlation analysis with Th1 or Tc9 cells. There was no significant correlation between the blood and pulmonary percentages of Th1 cells (Figure 5A) and between the blood and pulmonary Tc9 frequencies (Figure 5B) in the PCP group. The results, as shown in Figure 5C and 5D, indicate that the pulmonary Th1 cell levels in PCP patients exhibited an inverse correlation with the acute physiology and chronic health evaluation (APACHE) II score (*r* = -0.463, *P* = 0.0261) and sequential organ failure assessment (SOFA) score (*r* = -0.431, *P* = 0.0401). Interestingly, the frequency of lung Tc9 cells in the PCP cohort positively correlated with the APACHE II score (*r* = 0.4605, *P* = 0.0270; Figure 5E) and SOFA score (*r* = 0.4749, *P* = 0.0220; Figure 5F). Collectively, these results associated the host protective immune responses with the expansion of pulmonary Th1 cells and detrimental immune system activity with the expansion of pulmonary Tc9 subsets in patients with PCP.

## Discussion

PCP is a challenging complication in immunocompromised patients due to the severity of symptoms and high mortality rates among those affected. Recently, *Pneumocystis* colonization has been found with increasing frequency in patients with chronic obstructive pulmonary disease, contributing to the impairment of pulmonary function 30, 31. Although extensive research has been carried out on the role of CD4^+^ T cells in mice, no single study exists which characterizes the response of either CD4^+^ or CD8^+^ T-cell subsets in patients with PCP 32. As reported, Struijk GH et al. have measured levels of Th1, Th2 and Th17 subsets in blood, in the PCP cohort of a group of renal transplant patients, and revealed no difference between them and the control cohort 25. Recently, Th9 cells have been implicated in promoting pulmonary pathogenesis in a mouse model of *Pneumocystis* infection 21. Therefore, on the basis of the present data underscoring the strong involvement of T-cells in response to PCP, we present here an analysis of T-cell profiles in the circulation and in the lungs.

The present study provides a comprehensive assessment of the relative proportions of T-cell subsets in the circulation and in the lungs of immunocompromised patients with and without PCP, presenting an extensive characterization of T-cell patterns in local and systemic sites. Of note, we provide evidence here that blood T-cell subsets do not differentiate cohorts with and without PCP, but pulmonary Th1 and Tc9 appear to be significantly higher in patients with PCP than in those without. Thus, it might suggest that PCP blood phenotype did not parallel the T-cell activation pattern in the lungs. Our findings are thus in line with a previous study that reported no difference in Th1, Th2, and Th17 populations in the blood in patients with PCP following renal transplantation compared with control patients 25. To our knowledge, our data represent the first investigation into immune activation of pulmonary T-cell subsets in patients with PCP. These findings provide better understanding of the pathogenesis of PCP, and provide a significant clinical basis for studying host adaptive immunity in non-HIV immunocompromised patients with PCP.

In our results, a similar reduction in CD4^+^ T cells and increased prevalence of CD8^+^ T cells occurs in blood and BAL fluid in the cohort with PCP relative to the cohort without PCP. This disagrees with the previously reported finding that the circulating CD4^+^ T-cell counts in PCP patients were significantly reduced compared to those in control patients 25. We also failed to see a difference in circulating T-cell subsets between patients with and without PCP, suggesting similar immunosuppression of peripheral T-cell immune activation. Although only a trend was observed toward higher T-cell mediated production of IL-4 in BAL fluid from PCP patients, its levels in PCP patient blood showed no significant differences than in control patients, suggesting it has distinct roles in circulation and in the lungs. It has recently been proposed that Th2 mediated immune responses are enhanced in* P. murina*-infected animals, where they play a beneficial role in immunity 33. Further studies are needed to better characterize the role of Th2 in patients with PCP. Additionally, some studies have reported the significant roles of CD4^+^ T-cell derived IL-22 and granulocyte-macrophage colony-stimulating factor in host defense against PCP 34-36; however, we are missing important immune response in our present study. Thus, some studies should be conducted to investigate the significance of these cytokines.

Th17 cells have been seen to increase and possibly aid in clearance of *Pneumocystis* in animals 37. Our findings revealed similar levels of circulating Th17 cells in patients with and without PCP, in accord with recently reported data which saw no significant difference in Th17 percentages between cohorts with and without PCP in a study of renal transplant patients 25. In spite of these findings, further studies are necessary to explore the role of Th17 in immunocompromised patients with and without PCP.

Our study revealed a significant increase in Th1 cells in BAL fluid in PCP patients in contrast to that of patients without PCP, suggesting involvement of these IFN-γ-producing T cells in the pulmonary inflammatory response, either in controlling or promoting. Previous studies have reported the size of IFN-γ-producing T cell populations in the lungs of *P. murina* infected animals 16. Our findings appear to be in accord with this previous data. Several other studies have reported that IFN-γ-producing T cells are required for *Pneumocystis*-driven pulmonary inflammation. Paradoxically, Balb/c Stat4^-/-^ mice displayed susceptibility to *P. murina* infection in the lungs, while mice deficient in either IFN-γ or IL-4 remain able to resolve infection 38, 39. This discrepancy in experimental mice model studies might indicate the role of pulmonary Th1 cells is dispensable. Without consistence with these studies, we did find an adverse correlation between Th1 cell frequency and disease severity in the PCP patients, suggesting that pulmonary Th1 cells might play a protective role. By contrast to the percentages of IFN-γ-producing T cells in blood, we observed increased IFN-γ-producing T cells in BAL fluid in patients with PCP; thus, low circulating levels might be due to the increased migration into the lungs.

Recently, a great deal of attention has focused on the response of specific T- cell subsets, like Th1 40, Th2 18, Tregs 41, 42, and Tc 24 to PCP. In patients with PCP, the host immune response appears to be mediated not only by CD4^+^ T cells, but also by CD8^+^ T cells, which may moderate CD4^+^ cell-mediated pathology by elevating Treg percentages in mice 14. Several lines of evidence, suggest that the Tc1 subset of CD8^+^ T-cells compromises the host defense against *Pneumocystis* 23, 24. Contrary to those results, our data revealed enrichment of Tc9 subsets in the BAL fluid of PCP patients relative to that of patients without PCP. The most obvious finding to emerge from this study is that the Tc9 percentage in BAL fluid positively correlated with the disease severity in patients with PCP, implying that their contribution to immunopathogenesis is detrimental to PCP patients. Although we did not find a significant correlation between Tc1 prevalence and disease severity in the PCP cohort, these data corroborate the conclusions of previous studies, which suggest that Tc1 CD8^+^ T cells are not a primary contributor to lung damage 24. Indeed, the PCP patient specific expanded pulmonary Tc9 cell population could be targeted in the future to treat PCP in human patients.

IL-9 is reported to have a defect in patients with chronic mucocutaneous candidiasis 43, but its role in PCP remains unclearly defined. Increased IL-9 levels and Th9 cells have recently been seen in *P. murina*-infected mice, where their inverse correlation with clearance of *P. murina* suggested a role in pathogenesis 21. However, no studies have investigated the role(s) of Th9/Tc9 in the circulation or lungs of patients with PCP. In this context, our results suggest that a Tc9-related rather than a Th9-related response, is likely to perform a significant detrimental function in PCP cohorts. Together, these observations suggest that Th9 cell expansion plays an immunopathogenic role in humans as is observed in mice, and provide insight into the role of Tc9 cells in PCP.

Our observations presented here are intriguing in the circulating and pulmonary T-cell immune responses in HIV-negative patients with PCP. Furthermore, in our study, only pulmonary Th1 and Tc9 could distinguish patients with and without PCP. We did not find any correlation between the pulmonary Th1 or Tc9 frequencies and the circulating compartments in patients with PCP. These results are supported by earlier studies in which blood T-cell populations did not correlate with those in BAL fluid of HIV-negative patients with PCP 27. However, our data contrasts with data from a study of HIV-positive patients with PCP, where a correlation was found between T-cell populations in alveoli and blood 27, 44. Thus, our findings suggest that local T cells in the lungs might not reflect systemic events in the circulations. Mitigating the value of this discovery, acquisition of BAL fluid samples is a relatively invasive procedure to perform for diagnostic purposes, thus less invasive methods or predictive factors are needed.

We acknowledge some possible weaknesses are present in our study. With a small sample size and single study center, caution must be applied, as the findings might not be accurately representative. Another note of caution is due here since we are limited to subjects that were admitted to the ICU. Since our study was limited to Giemsa and methenamine silver staining of *Pneumocystis* using BAL fluid samples, which were known to have low sensitivity for detection of *Pneumocystis* and have the potential to be positive in patients with colonization, it was possible to misclassify some patients. Additional studies will be warranted to dissect the response mechanisms of specific subsets of T cells thoroughly. Further studies, which take these factors into account, will need to be undertaken.

## Conclusions

In summary, the data in this study complements data from animal studies and expands the available human data, and indicates that local expansion of Th1 and Tc9 subsets may play protective and detrimental roles in PCP, respectively. More broadly, the combination of findings presented here are assumed to have relevant implications for better understanding of T-cell responses, which may support development of immunotherapies aimed at specific subsets of Th1 or Tc9 cells for *Pneumocystis* infection.

## Figures and Tables

**Table 1:** Summary of patient characteristics

Characteristic	non-PCP (n =17)	PCP (n = 27)	*P* value
Demographics			
Age (years), mean ± SD	61.94 ± 13.07	57.148 ± 15.41	0.294
Gender, male, n (%)	11 (64.71)	14 (51.86)	0.402
Underlying diseases, n (%)			
Solid tumor	2 (11.76)	2 (7.41)	1.000
Hematological malignancy	1 (5.88)	3 (11.11)	0.961
Solid organ transplantation	1 (5.88)	2 (7.41)	1.000
Systemic disease	8 (47.06)	9 (33.33)	0.363
Interstitial lung disease	5 (29.41)	13 (48.15)	0.218
Immunosuppressive agents use, n (%)			
Corticosteroids	14 (82.35)	25 (92.59)	0.579
Anti-tumor chemotherapy	1 (5.88)	4 (14.81)	0.674
T cell immunosuppressant^a^	7 (41.18)	18 (66.67)	0.096
Prophylaxis at the time of the study	4 (23.53)	3 (11.11)	0.501
Laboratory findings			
White blood cells (10^9^/L), median (IQR)	7.05 (6.08-11.48)	10.23 (6.28-12.19)	0.286
Lymphocytes (10^9^/L), median (IQR)	0.36 (0.21-0.73)	0.54 (0.35-1.01)	0.339
CD4^+^ (cells/μL), median (IQR)	168 (90-293)	197 (79.5-297.5)	1.000
CD8^+^ (cells/μL), median (IQR)	154 (106-327)	220 (100-297.5)	0.6038

Note:^ a^The immunosuppressant included cyclosporine, mycophenolate mofetil, tacrolimus, programmed death-1 blocker, specific monoclonal antibodies (such as rituximab), and nucleoside analogues. Continuous variables are presented as median with IQR (25%, 75%) or mean ± SD. Other values are presented as numbers (%). PCP, *Pneumocystis jirovecii* pneumonia; SD, standard deviation; IQR, interquartile range.* P* < 0.05 was considered statistically significant.

## Abbreviations

HIV: human immunodeficiency virus
PCP: *Pneumocystis jirovecii* pneumonia
ICU: intensive care units (ICU)
HC: healthy controls
BAL: bronchoalveolar lavage
PBMCs: peripheral blood mononuclear cells
ICS: intracellular cell staining
PMA: phorbol myristate acetate
SD: standard deviation
IQR: interquartile range
APACHE: acute physiology and chronic health evaluation
SOFA: sequential organ failure assessment
